# RNA Sequencing Reveals the Regulation of Betaine on Chicken Myogenesis

**DOI:** 10.3390/ani12192508

**Published:** 2022-09-21

**Authors:** Zhijun Wang, Danfeng Cai, Xing Ju, Kan Li, Sisi Liang, Meixia Fang, Qinghua Nie

**Affiliations:** 1 Department of Animal Genetics, Breeding and Reproduction, College of Animal Science, South China Agricultural University, Guangzhou 510642, China; 2 Guangdong Provincial Key Lab of Agro-Animal Genomics and Molecular Breeding and Key Laboratory of Chicken Genetics, Breeding and Reproduction, National-Local Joint Engineering Research Center for Livestock Breeding, Ministry of Agriculture, Guangzhou 510642, China; 3 Department of Laboratory Animal Science, Medical College of Jinan University, Guangzhou 510632, China

**Keywords:** betaine, myogenesis, N6-Methyladenosine, mRNA-Seq, chicken

## Abstract

**Simple Summary:**

Betaine is a healthy source of methyl and glycine and a widely used feed additive to promote animal growth. Previous studies mainly focused on its anti-osmotic pressure, anti-inflammatory, and growth-promoting effect in vitro. Still, poultry’s growth-promoting mechanism and regulation of muscle cells remain unknown. In this study, we found that a low concentration of betaine could inhibit myoblast cell proliferation but promote myotube formation, suggesting that the growth-promoting effect of betaine was achieved through the differentiation and fusion of myotubes. In addition, RNA sequencing found a series of betaine-affected genes, which could provide a theoretical basis for explaining betaine’s regulation on chicken myogenesis in vitro.

**Abstract:**

Betaine is trimethylglycine and a universal methyl donor which could provide methyl and glycine for cells and animals. As a new star in epigenetics, N6-Methyladenosine has been reported to regulate multiple biological activities, but the regulatory mechanism of betaine on N6-Methyladenosine as well as myogenesis was little studied. In this study, we treated chicken primary myoblast cells with different concentrations of betaine (0, 10, 25, and 50 mmol/L) and found that myoblast cell proliferation was inhibited, although the cell cycle was promoted in the S phase by betaine, where the myotube area was increased as well as the differentiation marker genes *MyoD*, *MyoG*, *MyHC*, *Myomarker*, and *Ckm*. RNA sequencing obtained a total of 61 differentially expressed genes (DEGs); DEGs caused by 50 mmol/L betaine were mainly enriched in the regulation of skeletal muscle tissue regeneration and some amino acid metabolic processes. The gene expression pattern trends of all DEGs were mainly clustered into 2 profiles, with the increase in betaine concentration, the gene expression pattern either increased or decreased continuously. Overall, a low concentration betaine can increase the N6-Methyladenosine modification level and myotube area but depresses myoblast cell proliferation in vitro.

## 1. Introduction

Betaine is known as trimethylglycine (a glycine with three methyl groups), which could produce the universal methyl donor in transmethylation for homocysteine to form S-adenosyl methionine (SAM) or degrade resulting in the formation of trimethylamine and glycine [1,2,3]. The DNA- and RNA-methyltransferases could catalyze SAM to provide a methyl group for DNA and RNA bases thus completing the methylation of DNA and RNA bases [4,5]. For example, the RNA base A could directly attack the SAM coenzyme and lead to immediate RNA methylation [6]. The demethylation of SAM could transform SAM into S-adenosyl homocysteine (SAH), so the ratio of SAM: SAH could affect a series of SAM-dependent methyltransferases such as phosphatidylethanolamine methyltransferase and protein arginine methyltransferase, as well as DNA- and RNA-methyltransferases [3,6,7,8].

N6-Methyladenosine (m^6^A) is a common type of RNA methylation; it has been 10 years since m^6^A was identified as a reversible chemical modification [9,10], and is the first reversible RNA modification that occurs on adenosine that has ever been found [11,12]. m^6^A has been reported to play important roles in various biological activities such as mouse embryonic cell differentiation [13], yeast meiosis [14], and many cancer progressions [15]. In poultry, studies have shown that m^6^A participates in ovary follicle selection [16], adipose deposition [17], primordial germ cell formation [18], and gonadal sex differentiation [19]. The growth and development of muscle are also inseparable from the modification of RNA methylation. Research has shown that the methyltransferase METTL3 could regulate myoblast transition [20] and muscle maintenance and growth [21].

Betaine as a methyl donor has been reported to regulate the differentiation of murine myoblasts via IGF-1 signaling [22]. Previous in vivo studies on chicks [23,24], pigs [2,25], and ruminants [1] have confirmed that betaine could be used as a feed additive to provide methyl in methionine-deficient diets and promote animal growth rates. However, most research was carried out after birth, and the regulatory mechanism of betaine on chicken primary myoblasts and m^6^A level was little studied. Thus, in this study, we treated chicken primary myoblast cells with different concentrations of betaine to detect the changes in their m^6^A levels and the effects on myotube formation.

## 2. Materials and Methods

### 2.1. Ethics Statement

The chicken primary myoblast isolation protocol was handled in compliance and approved by the Institutional Animal Protection and Utilization Committee of South China Agricultural University.

### 2.2. Isolation and Culture of Chicken Primary Myoblast Cells

The isolation protocol of chicken primary myoblast cells was followed as previously described [26]. Cells for proliferation assay were cultured in growth medium (GM) (RPMI-1640 medium with 20% fetal bovine serum and 0.5% penicillin/streptomycin (Gibco, Carlsbad, CA, USA)). For differentiation assay the medium was replaced with differentiation medium (DM) (RPMI-1640 medium with 5% horse serum and 0.5% penicillin/streptomycin).

### 2.3. Betaine Treatment and RNA Dot Blot

Different concentrations of betaine (Sigma, St. Louis, MO, USA) were diluted in a growth medium or differentiation medium and passed through a 0.22 μm filter. Different concentrations of betaine (0, 10, 25, 50, 100, 200 mmol/L) were added into 12-well plates (3 wells per treatment) or 96-well plates (6 wells per treatment) three times to treat cells in the proliferation and differentiation phase for 24 h, except CCK8 assay treat for 24, 36, and 48 h.

RNA dot blot was performed by using 2 μg total RNA, RNA was added to the nylon membrane after being denatured at 95 °C for 3 min. RNA was crosslinked to the membrane with 254 nm 125 mJoule/cm^2^ UV light for 60 s. The membrane was washed in TBST for 5 min, blocked in 5% silk milk in TBST for 1 h, and incubated overnight with N6-Methyladenosine primary antibody (1:500, #56593, Cell Signaling Technology, Beverly, MA, USA). After washing three times with TBST, the membrane was then incubated with HRP conjugated goat- anti-rabbit IgG (1:10,000, Abbkine, Wuhan, China) for 1 h. A Licor Odyssey (Licor, Lincoln, NE, USA) was used to capture the image of the membrane. The same RNA was crosslinked to the nylon membrane and stained with 0.1% methylene blue solution for 30 min and washed with TBST 3 times for 10 min to measure the total nucleic acid level.

### 2.4. RNA Isolation, cDNA Synthesis and Quantitative Real-Time PCR (qRT-RCR)

Total RNA was extracted with RNAiso Plus reagent (Takara, Otsu, Japan) using the chloroform isopropanol method. HiScript II Q RT SuperMix for qPCR (+ gDNA wiper) (Vazyme, Nanjing, China) was used for cDNA synthesis, and then cDNA was used for qRT-PCR with iTaqTM Universal SYBR Green Supermix (Bio-Rad, Hercules, CA, USA). The primers used for qRT-PCR are listed in Table S1.

### 2.5. Proliferation Assay

EdU assay: EdU (5-Ethynyl-2′-Deoxyuridine) assay was performed with a C10310 EdU Apollo In Vitro Imaging Kit (Ribobio, Guangzhou, China) after myoblasts were incubated in 50 μmol/L 5-ethynyl-2′-deoxyuridine for 2 h after 22 h betaine treatment. All images were captured with a Leica DMi8 fluorescent microscope (Leica, Wetzlar, Germany) in 200× with 6 random fields in 3 wells per group. The proliferation rate = (EdU positive cells)/(total Hoechst 33,342 stained cells)

Cell cycle analysis: myoblasts were harvested after 24 h betaine treatment and fixed in 70% ethanol overnight at −20 °C. Then, the cells were incubated with 50 μg/mL propidium iodide (Sigma, St. Louis, MO, USA), 10 μg/mL RNase A (Takara, Otsu, Japan), and 0.2% Triton X-100 (Sigma, USA) for 30 min at 4 °C. Cell cycle analysis was performed with a BD AccuriC6 flow cytometer (BD Biosciences, USA) and FlowJo (v7.6) software (Treestar Incorporated, Woodburn, OR, USA).

CCK-8 Assay: CCK-8 (Cell Counting Kit 8) assay was performed in a 96-well plate with 10 μL CCK solutions and incubated for 1 h in the cell incubator after 23 h, 35 h, and 47 h betaine treatment. A Fluorescence/Multi-Detection Microplate Reader (BioTek, Winooski, VT, USA) was used here to measure the absorbance at the wavelength of 450 nm.

### 2.6. Differentiation Assay

The chicken primary myoblast cells were digested with trypsin and seeded at a density of 2 × 10^4^ cells/cm^2^ on 12-well culture plates with GM. The medium was replaced with DM after 12 h to induce myoblast differentiation, and we defined this day as day 1 (DM 1). The betaine was added on day 3, and the cells were fixed and stained after 24 h. Chicken primary myoblasts were fixed with 4% formaldehyde and blocked in 0.2% Triton-X 100 and 5% horse serum with PBS for 30 min, and incubated overnight with MF20 (1:100, Developmental Studies Hybridoma Bank, Iowa City, IA, USA) primary antibody. After washing three times with PBS, the cells were incubated with goat anti-mouse IgG (H+L)-Dylight 594 (1:200, BS10027; Bioworld, Minneapolis, MN, USA) and Hoechst 33,342 (1 mg/mL, H1399, Invitrogen, Waltham, MA, USA) for 1 h the next day. The cultures were rinsed with PBS 3 times and left in PBS for imaging. All images were captured with a Leica DMi8 fluorescent microscope (Leica, Wetzlar, Germany) in 200× with 6 random fields in 3 wells per treatment. The fusion index (the number of nuclei inside myotubes/total number of nuclei) and the percentage of myotube area were calculated using ImageJ software (National Institutes of Health, Bethesda, MD, USA).

### 2.7. RNA Sequencing

The total RNA of myoblast cells treated with betaine (0, 10, 25, 50 mmol/L) was extracted and sent to Gene Denovo Biotechnology Co. (Guangzhou, China) for cDNA library construction and sequencing using Illumina Novaseq6000 platform and named Bet_0, Bet _10, Bet _25, Bet _50. The standard of differentially expressed genes (DEGs) was set as |(Fold change)| ≥ 1.5 and FDR < 0.05. Bioinformatic analysis was performed using Omicsmart, a dynamic real-time interactive online platform for data analysis (http://www.omicsmart.com accessed on 1 June 2022). In addition, trend analysis was performed for all DEGs to examine the expression pattern of DEGs. The expression data of each sample (in the order of treatment) were clustered using Short Time-series Expression Miner software (STEM), and the clustered profiles with a *p*-value ≤ 0.05 were considered significant profiles [27,28]. All the sequence data have been deposited in CNCB (China National Center for Bioinformation) Genome Sequence Archive (GSA, https://ngdc.cncb.ac.cn/gsub/, accessed on 1 June 2022) and are accessible through GSA series accession number CRA006598: https://ngdc.cncb.ac.cn/gsa/s/M5Mdoj9M (accessed on 1 June 2022).

### 2.8. Statistical Analysis

The relative expression of all detected RNA at differentiation concentrations was compared with 0 mmol/L and calculated using the 2 (-Delta Delta C(T)) method [29]. The statistically significant difference between 0 mmol/L and other concentrations was tested using an independent sample *t*-test. All results were presented as mean ± S.E.M and repeated three times with three independent wells each time. We considered *p* < 0.05 to be statistically significant. * *p* < 0.05; ** *p* < 0.01; *** *p* < 0.001.

## 3. Results

### 3.1. Betaine Inhibits Myoblast Cell Proliferation In Vitro

**Figure 1:**
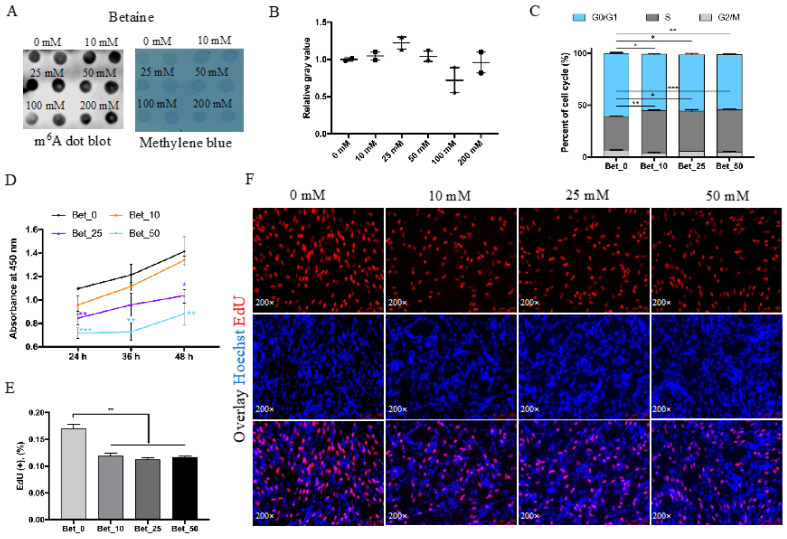
Betaine inhibits myoblast cells proliferation. (**A**) RNA dot blot and methylene blue results after being treated with 0, 10, 25, 50 mmol/L betaine for 24 h. (**B**) The relative gray value of RNA dot blot. (**C**) Cell cycle analysis of different cell stages after 24 h betaine treatment. (**D**) The statistical results of CCK-8 assay after 24 h, 36 h, and 48 h betaine treatment. (**E**) The EdU positive cell rate (**F**) and EdU staining of myoblast cells being treated with betaine. (mM refers to mmol/L; * *p* < 0.05; ** *p* < 0.01; *** *p* < 0.001).

To investigate the effect of betaine on N6-Methyladenosine levels, we treated myoblast cells with different concentrations of betaine. The m^6^A level was successfully increased after being treated with 10, 25, and 50 mmol/L betaine for 24 h but decreased with 100 and 200 mmol/L betaine (Figure 1A,B). Therefore, we wanted to study the effect of betaine on myoblast proliferation and differentiation in the presence of increased methylation levels by using 0, 10, 25, and 50 mmol/L betaine for our study. The cell cycle analysis results showed that the number of cells in the S phase significantly increased with the increased concentration of betaine along with decreased cells in the G0/G1 phase (Figure 1C). The results of the CCK8 experiment showed that betaine inhibited myoblast cell proliferation, and the longer the treatment time, the more significant the inhibitory effect was (Figure 1D). EdU positive cells (Figure 1E) and EdU staining (Figure 1F) results showed that myoblast cells in the proliferative stage decreased after 24 h of betaine treatment.

### 3.2. Betaine Promotes Myotube Formation In Vitro

**Figure 2:**
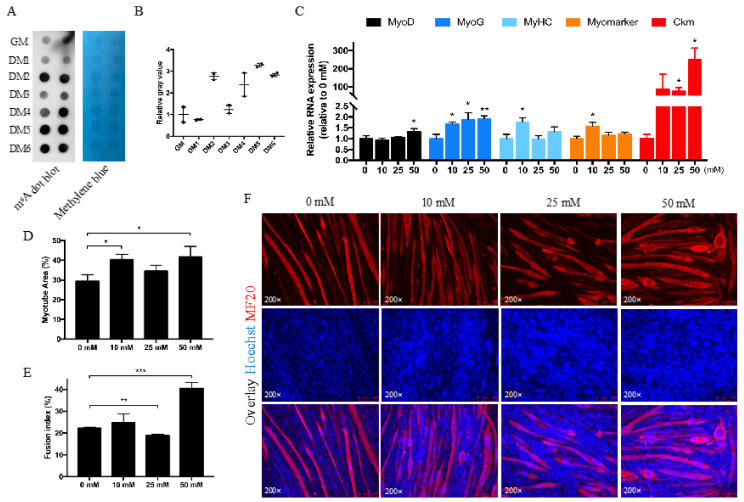
Betaine promotes myotube formation. (**A**) RNA dot blot and methylene blue results during myoblast differentiation. (**B**) The relative gray value of RNA dot blot. (**C**) qRT-PCR results for *MyoD*, *MyoG*, *MyHC*, *Myomarker*, and *Ckm* in myoblast cells after being treated with 0, 10, 25, 50 mmol/L betaine for 24 h. (**D**–**F**) Myotube area (%) analysis (**D**), fusion index € of MF20 immunofluorescence staining (**F**) after being treated with 0, 10, 25, 50 mmol/L betaine for 24 h at DM3. (mM refers to mmol/L; * *p* < 0.05; ** *p* < 0.01; *** *p* < 0.001).

The m^6^A level was dynamic during myoblast differentiation, which is relatively low in the proliferation phase (GM) and the first day of differentiation (DM1), increases on the second day of differentiation (DM2), then decreases on the third day (DM3), and then increases continuously (Figure 2A,B). We treated cells in DM3 with betaine to increase the m^6^A level to check the influence of betaine on myotube formation. The results showed that betaine could promote the formation of myotubes with a 10 to 50 mmol/L concentration, and the concentrations of 10 and 50 mmol/L have a better effect than other concentrations (Figure 2D–F). The mRNA level of MyoG, MyHC, and Myomarker were significantly increased in the 10 mmol/L betaine treatment group, and in 50 mmol/L concentration, MyoD, MyoG, and Ckm (muscle creatine kinase) were significantly promoted (Figure 2C). Although the fusion index of 25 mmol/L was slightly lower than control (Figure 2D), the Ckm level was dramatically up-regulated, suggesting the promotion of muscle differentiation. 50 mmol/L betaine treatment significantly promoted myotube area (Figure 2D), fusion index (Figure 2E), and differentiated myotubes formation (Figure 2F). These data suggest that betaine treatment promotes muscle differentiation in vitro.

### 3.3. RNA-Sequencing Analysis for DEGs in Betaine-Treated Myoblast Cells

**Figure 3:**
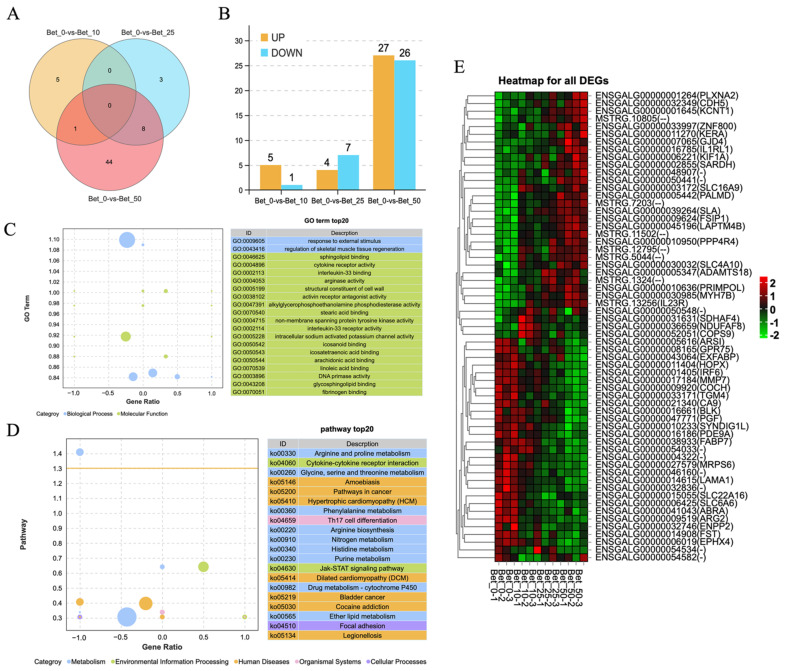
Overview of mRNA sequencing in betaine-treated myoblast cells. (**A**) Venn diagram shows common and unique DEGs between groups. (**B**) The number of differentially up- and down-expressed genes in each group. (**C**) GO functional enrichment analysis of DEGs in Bet_0 vs. Bet_50. (**D**) KEGG pathway analysis of DEGs in Bet_0 vs. Bet_50. (**E**) Heatmap for all 61 DEGs.

A total of 12 samples from the myoblast cells with 3 replications per betaine treatments (0, 10, 25, and 50 mmol/L) were collected for mRNA-Seq. All raw data were submitted to the CNCB GSA database (accession number CRA006598). In the mRNA-Seq results, we acquired at least 38.61 million clean data points and 5.76 billion clean bases for each sample. The clean reads were mapped to chicken GRCg6a (Ensembl_release100) and detected a total of 17,355 genes with 16,666 known genes and 689 novel genes (Table S2). Compared to the Bet_0 control group, other treatment groups caused a total of 61 DEGs; there were small amounts of overlapping DEGs among different treatment groups, but no DEGs were commonly differentially expressed in three treatment groups (Figure 3A). The number of DEGs increases with the increase in concentration—6 DEGs in Bet_10, 11 DEGs in Bet_25, and 53 DEGs in Bet_50 (Figure 3B). The gene ontology (GO) functional enrichment of DEGs between Bet_0 and Bet_50 showed that the enriched biological process was a response to external stimuli and regulation of skeletal muscle tissue regeneration. The enriched molecular functions were mainly sphingolipid binding, cytokine receptor binding, and so on (Figure 3C, Table S3). The KEGG (Kyoto Encyclopedia of Genes and Genomes) pathway analysis showed that the DEGs in the Bet_50 group were mainly enriched in amino acid metabolism pathways and some human diseases pathways (Figure 3D, Table S4). The heatmap of all DEGs (Table S5) is shown in Figure 3E, where the expression went higher from green to red, so the top 32 genes were betaine-related up-regulated genes, and the bottom 29 genes were betaine-related down-regulated genes. The heatmap results showed that the gene expression pattern was different between groups while similar intra-group (Figure 3E).

### 3.4. Trend Analysis of Gene Expression for Betaine Treatment

**Figure 4:**
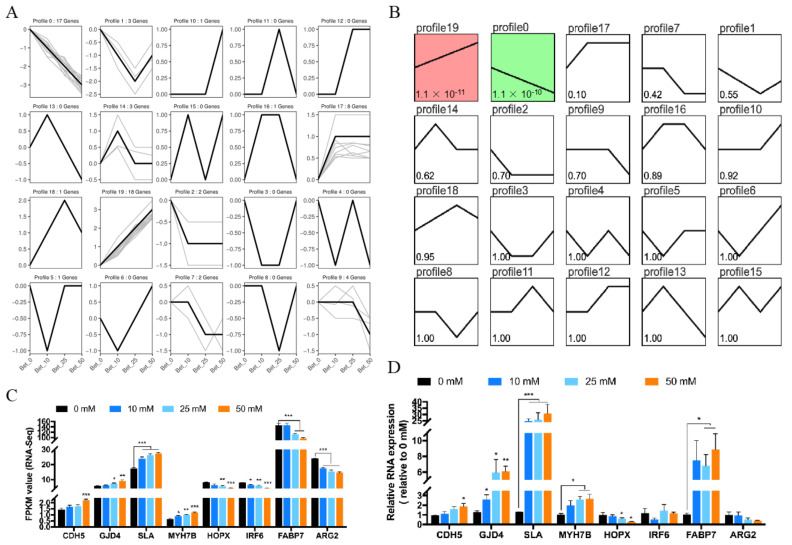
Gene expression pattern analysis of all DEGs. (**A**) General chart of all gene expression trend profiles. (**B**) Gene expression trend profiles are ordered based on the *p*-value significance of the number of genes assigned versus expected. (**C**, **D**) the FPKM value (**C**) and qRT-PCR validation (**D**) of eight DEGs in profile 0 and 19. (mM refers to mmol/L; * *p* < 0.05; ** *p* < 0.01; *** *p* < 0.001.)

Trend analysis, also known as gene expression pattern analysis, is used to cluster genes of similar expression patterns for multiple samples. To confirm whether betaine-treated myoblast cells were dose-dependent, we performed a trend analysis for all 61 DEGs; altogether, there could have been 20 expression profiles, and our DEGs covered 12 profiles (Figure 4A, Table S6), but only 2 profiles (profile 0 and 19) were clustered significantly (Figure 4B, Table S7). Among those two profiles, the expression of 17 genes in profile 0 was consistently decreased while in profile 19, the expression of 18 genes was consistently increased, proving that more than half (35/61) of the DEGs were dose-dependent. Four genes were randomly selected from profile 19 (CDH5, GJD4, SLA, and MYH7B) and profile 0 (HOPX, IRF6, FABP7, and ARG2) to validate the RNA-Seq results. Except for the expression of IRF6 and FABP7, the expression of the other 6 DEGs were consistent with the RNA-Seq results (Figure 4C,D), indicating that the RNA-Seq results were reliable.

## 4. Discussion

Previous studies showed that betaine could participate in the methionine-homocysteine cycle [30]. Research has found that the limitation of SAM could trigger cell cycle arrest in G1 [31], and therefore, when there is enough betaine in the cell to form SAM, cell cycle progression was promoted, from G0/G1 to the S phase (Figure 1C). The cell cycle results were consistent with previous studies with the exception of the CCK8 and EdU assay results, which were contrary to expectations. As CCK8 assay could not only detect cell proliferation, but also reflect cytotoxicity to a certain extent, judging from the CCK8 and EdU assay results, it is possible that the myoblasts were too fragile to resist the toxicity of betaine, thus resulting in fewer cells in the proliferation period in the EdU staining assay. From the perspective of cell density, with the same initial cell amount, unfused myoblast cells were promoted by betaine treatment after induced differentiation. This suggests myoblast cells in the differentiation phase were strong enough to overcome the toxicity of betaine, which means after myoblasts were induced to differentiate, the myoblasts were promoted in number and fusion. In vivo studies proved that betaine could be used as a feed additive to provide methyl in methionine-deficient diets and promote animal growth rates [1,2,23,24,25]; in this study, we showed that betaine could promote the expression of *MyoD*, *MyoG*, *MyHC*, *Myomarker*, and *Ckm* as well as myotube formation (Figure 2C–E). Our results indicated that with the addition of betaine and the increased m^6^A level, the differentiation of myoblast cells became more active.

Despite betaine being known as a universal methyl donor, we believe that the increase in m^6^A level may only require betaine concentrations up to 50 mmol/L (Figure 1A). However, the myogenesis process still increased significantly at 100 mmol/L, although it was not significant at 200 mmol/L (the results of MF20 staining at 100 and 200 mmol/L were not shown in Figure 2), which is different from previous reports that the m^6^A level increased 5 times at 0.2 mmol/L concentration in murine myoblasts [32]. Research has found that betaine could enhance C2C12 differentiation at 10 mmol/L concentration through the IGF1 pathway [22]. Our results indicated that, unlike murine myoblasts, 10 and 25 mmol/L betaine did not cause many DEGs in poultry myoblast cells (Figure 3A,B), and instead of the IGF1 pathway, the Jak-STAT signaling pathway was enriched in 50 mmol/L betaine treatment (Figure 3D), which means the regulation mechanism of betaine on myoblast cells in poultry and livestock may be different. The activation of the Jak-STAT signaling pathway could promote not only muscle differentiation [33] and hypertrophy [34,35], but also muscle regeneration [36]. The two differentially up-regulated genes (ENSGALG00000050441-CD131 and MSTRG.13256-IL23R) were enriched into the Jak-STAT signaling pathway, implying that betaine may regulate the myoblast differentiation through the Jak-STAT signaling pathway.

In addition to m^6^A modification and the IGF1 pathway, betaine may have other mechanisms to regulate myogenesis which lead to the decrease in m^6^A level after 100 mmol/L. A large amount of methyl accumulation will not continuously lead to the up-regulation of RNA methylation levels in chicken myoblast cells. As a methyl donor, extra methyl caused by betaine in myoblasts may also affect the methylation of DNA, another post-synthesis chemical modification [37]. Research showed that the DNA methylation level of muscle tissue in the elderly was significantly higher than that in the young [38]. In poultry, the differential modification of DNA methylation level may also lead to the difference of muscle growth rate [39]. Betaine affects not only RNA methylation but also DNA methylation, and this may explain why there was a small decrease in myotube formation at 25 mmol/L treatment.

## 5. Conclusions

The proliferation and differentiation of myoblast cell before birth greatly affect the muscle production after birth. Here, in this study, we found that a low concentration of betaine in chicken myoblast cell could inhibit the proliferation of myoblast cell but promote myotube formation, where half of the differentially expressed genes caused by betaine were dose-dependent. All of our results provide a theoretical basis for explaining betaine’s regulation on chicken myogenesis in vitro.

## Data Availability

The original data in the article can be obtained directly from the corresponding author. All the sequence data have been deposited in CNCB (China National Center for Bioinformation) Genome Sequence Archive GSA, https://ngdc.cncb.ac.cn/gsub/ (accessed on 1 June 2022) and are accessible through GSA series accession number CRA006598: https://ngdc.cncb.ac.cn/gsa/s/M5Mdoj9M (accessed on 1 June 2022).

## Supplementary Materials

The following supporting information can be downloaded at: https://www.mdpi.com/article/10.3390/ani12192508/s1, Table S1: The primers used for qRT-PCR; Table S2: The raw data, basic reads, filter, and region information of mRNA-Seq in each sample; Table S3: Top 30 significantly enriched GO terms information in Bet_50 vs Bet_0; Table S4: Top 30 KEGG pathway information in Bet_50 vs. Bet_0; Table S5: All DEG information used for heatmap in Figure 3E; Table S6: All DEG profile information used for gene expression pattern analysis; Table S7: Profile model, gene number, and *p*-value for each profile.
